# Lymphocyte subpopulations in myocardial infarction: a comparison between peripheral and intracoronary blood

**DOI:** 10.1186/s40064-015-1532-3

**Published:** 2015-12-01

**Authors:** Natalia Lluberas, Natalia Trías, Andreína Brugnini, Rafael Mila, Gustavo Vignolo, Pedro Trujillo, Ariel Durán, Sofía Grille, Ricardo Lluberas, Daniela Lens

**Affiliations:** Flow Cytometry and Molecular Biology Laboratory, Facultad de Medicina, Hospital de Clínicas, Universidad de la República, Av. Italia s/n., Montevideo, 11600 Uruguay; Department of Cardiology, Facultad de Medicina, University Cardiovascular Center, Hospital de Clínicas, Universidad de la República, Montevideo, Uruguay

**Keywords:** Immune response, Lymphocytes, Acute coronary syndrome, Flow cytometry, CD4CD28null

## Abstract

The frequency and profile of lymphocyte subsets within the culprit coronary artery were investigated in 33 patients with myocardial infarction and compared to their systemic circulating counterparts. T cell subsets including CD4^+^CD28null, activated and regulatory T-cells, TH1/TH2/TH17 phenotypes, NK and B-cells were studied in intracoronary (IC) and arterial peripheral blood (PB) samples. CD4^+^CD28null T-lymphocytes were significantly increased in IC compared to PB (3.7 vs. 2.9 %, p < 0.0001). Moreover, patients with more than 6 h of evolution of STEMI exhibited higher levels of CD4^+^CD28null T-cells suggesting that this subset may be associated with more intense myocardial damage. The rare NK subpopulation CD3^−^CD16^+^CD56^−^ was also increased in IC samples (5.6 vs. 3.9 %, p = 0.006). CD4^+^CD28null T-cells and CD3^−^CD16^+^CD56^−^ NK subpopulations were also associated with higher CK levels. Additionally, IFN-γ and IL10 were significantly higher in IC CD4^+^ lymphocytes. Particular immune cell populations with a pro-inflammatory profile at the site of onset were increased relative to their circulating counterparts suggesting a pathophysiological role of these cells in plaque instability, thrombi and myocardial damage.

## Background

Atherosclerosis is regarded as a chronic inflammatory disease of the arterial wall (Hansson 2005). A large body of evidence supports the involvement of disregulated adaptive immunity in atherogenesis and its complications (Libby et al. 2009; Shah 2007; Matusik et al. 2012). Firstly, plaque instability has been associated with an increased numbers of intra-plaque activated T-cells (Jonasson et al. 1986) and with CD4^+^ T lymphocytes preferentially differentiated into Th1 cells (Martens et al. 1997; van der Wal et al. 1994). Secondly, postmortem studies showed that this CD4^+^ subset accumulates preferentially in ruptured plaques and produces high levels of Interferon-gamma (IFN-γ) (Eid et al. 2009; Giubilato et al. 2011; Liuzzo et al. 2000).

Additionally, detailed analysis of CD4^+^ T-cells in acute coronary syndromes (ACS) revealed the expansion in peripheral blood of an unusual subset (Morishita et al. 1989), that does not express the co-estimulatory receptor CD28 (CD4^+^CD28^null^) (Liuzzo et al. 1999) (Zal et al. 2008). Finally, reduction of natural killer (NK) and regulatory T-cells (Tregs) are linked to atherosclerosis (Jonasson et al. 2005; Han et al. 2007; Sardella et al. 2007). However, Ammirati et al. showed that Tregs were increased in STEMI compared with non-ST- elevation ACS (Ammirati et al. 2010).

Despite the clear links between these subsets and atherothrombosis, one relevant question is, to what extent the lymphocyte changes in peripheral blood reflect the immune response at the site of the onset.

Primary percutaneous coronary intervention (PCI) is the treatment of choice in STEMI (Antman et al. 2004). The use of an aspiration catheter, for which clinical benefit has been documented (Sardella et al. 2009, 2010), allows sampling human intracoronary blood during the acute coronary event at the site of the coronary occlusion.

In this work, we investigated the involvement of distinct cell subsets of the innate and adaptive immune response in patients with STEMI undergoing PCI as well as the expression of different pro- and anti-inflammatory cytokines at the site of the occlusion and compared them with its levels in systemic circulation, hypothesizing that coronary blood analysis may reveal changes in the immune response that are not detectable in peripheral blood.

## Results

### Characteristics of the study population

The general characteristics of the study participants are summarized in Table 1. There were 25 men and 8 women with a mean age of 62 ± 15 years. The mean interval from STEMI onset to reperfusion was 8 ± 7 h (range 1.5–24). The median value of the maximum CK levels was 1.672 U/l, IQR 1130-2524, and 61 % were anterior infarctions.

**Table 1:** Baseline Characteristics of Patients (n = 33)

Age (years)	62 ± 13
Female, n (%)	8 (24)
Smoking, n (%)	20 (60)
Hypertension, n (%)	18 (54)
Diabetes mellitus, n (%)	6 (18)
Hypercholesterolemia, n (%)	14 (42)
KK I, n (%)	26 (78)
CK (IU/L)	1672 (IQR 1130–1672)
Anterior infarction, n (%)	21 (61)
Pain to balloon time (hours)	8.81 ± 7.01
Pre PCI TIMI flow 0–1 grade, n (%)	30 (91)
Post PCI TIMI flow 3 grade, n (%)	31(96)
Myocardial blush 3, n (%)	31 (96)

### Lymphocyte subsets in intracoronary and arterial peripheral blood

#### T cells

No differences were found in total T cell frequency among the IC and PB samples (Table 2). The proportion of total CD3^+^, CD4^+^ and CD8^+^ T-cells were expressed as a percentage of total lymphocytes. The relative proportion of CD4^+^ and CD8^+^ T-cells among the CD3^+^ cell population, was similar for both IC and PB samples.

**Table 2:** Comparative analysis of frequencies of mayor lymphocyte subsets in arterial PB and IC blood

	IC blood	PB	p
	Median (IQR)	Median (IQR)	
T cells	67.7 (59.5–74.2)	64.8 (59.3–75.3)	NS
CD4^+^	57.7 (47.1–68.3)	57.7 (47.2–67.7)	NS
CD8^+^	36.1(28.1–47.1)	35.5 (26.5–46.1)	NS
CD4^+^CD69^+^	1.7 (0.6–7.5)	1.6 (0.4–4.6)	NS
CD8^+^CD69^+^	3.4 (2.1–19.2)	3.6 (1.8–14.3)	NS
CD4^+^CD28^null^	3.7 (0.9–8.5)	2.9 (0.5–6.6)	<0.0001
CD4^+^CD25^+^FoxP3^+^	5.5 (3.8–6.9)	6.1 (3.7–7.5)	NS
CD3^+^CD56^+^	5.8 (3.0–8.7)	7.1 (4.1–10.9)	NS
Total NK cells	14.7 (8.3–20)	14.5 (28.6–21.3)	NS
CD3^−^CD56^+^CD16^+^	88.2 (82–93.1)	89.6 (83.1–92.6)	NS
CD3^−^CD56^+^CD16^−^	4.8 (2.5–6.8)	3.5 (2.8–5.8)	NS
CD3^−^CD56^−^CD16^+^	5.6 (3.3–9.2)	3.9 (2.8–7.8)	0.006
B cells	10.3 (4.0–13.2)	11.0 (5.9–15.7)	0.008

Values are expressed as median and interquartile range (IQR). For total T cells, total NK cells, CD3^+^CD56^+^ T cells and B cells, values are given as percent of lymphocytes and for CD4^+^ and CD8^+^ T cells as a percentage of CD3^+^ cells. For NK subsets, values are presented as percent of NK cells. Regulatory T cells, CD4^+^CD28^null^ and CD4^+^CD69^+^ cells are expressed as a percentage of CD3^+^CD4^+^, and CD8^+^CD69^+^ as a percentage of CD3^+^CD8^+^ lymphocytes. Comparing IC and PB samples, there were significant differences in CD4^+^CD28^null^, CD3^−^CD56^−^CD16^+^ and B cell subpopulations

**Fig. 1:**
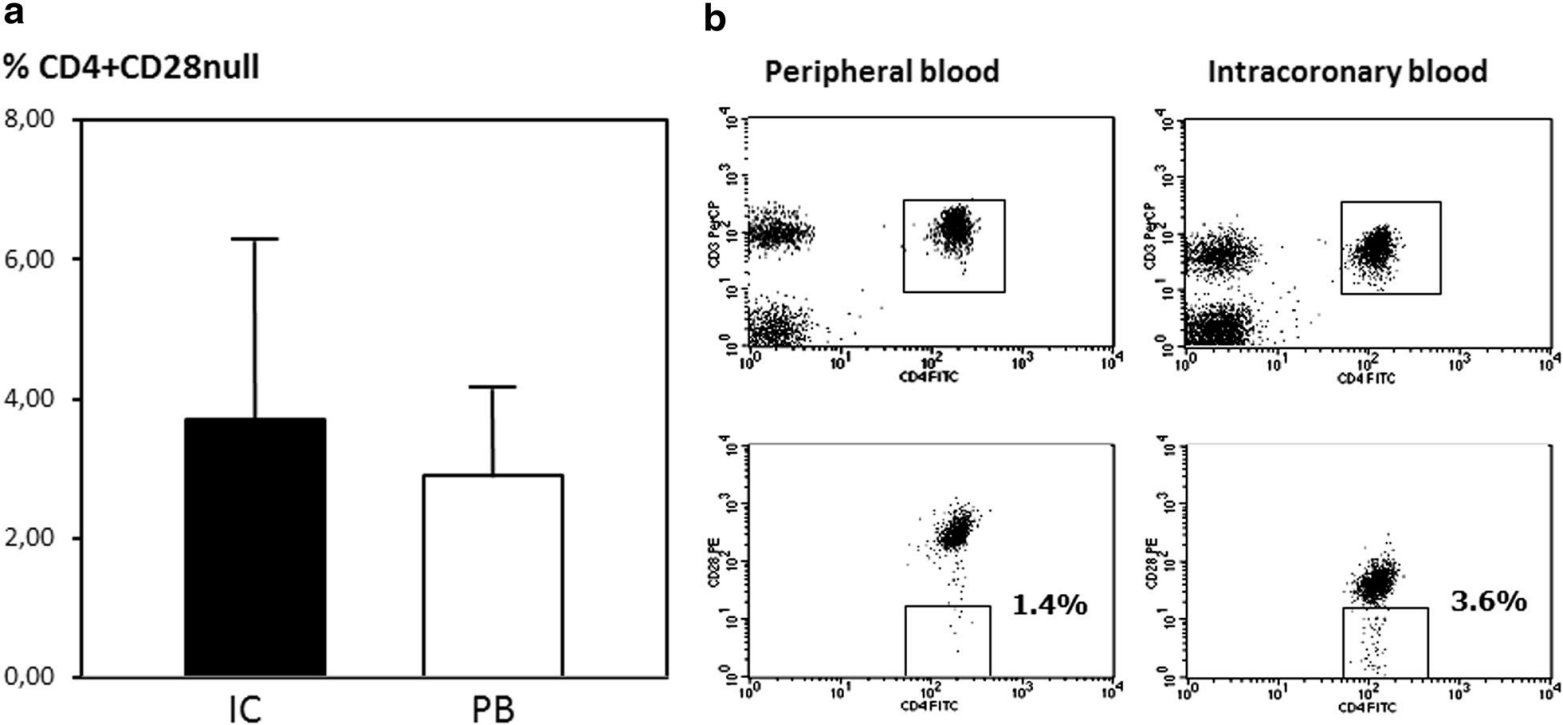
**a** Comparison of CD4^+^CD28^null^ T-cells in IC and PB samples. The frequency of CD4^+^CD28^null^ T-cells was significantly higher in IC blood (p < 0.001, Wilcoxon signed rank test). **b** Representative flow cytometry data from PB and IC lymphocytes showing the gating strategy for CD4^+^CD28^null^ T cell analysis. An initial region was performed in a FSC/SSC diagram to circumscribe the lymphocyte population (not shown) and a second region was defined for CD3^+^CD4^+^ cells. Thereafter identification of CD28^−^ cells was performed

**Fig. 2:**
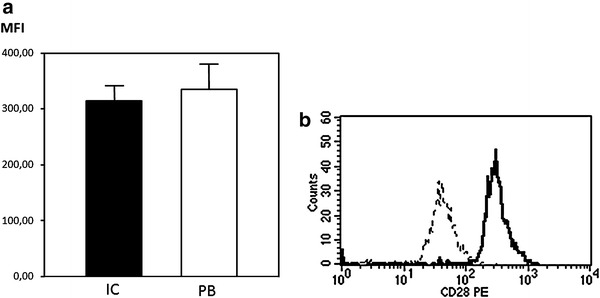
**a** Mean fluorescence intensity (MFI) of CD28 in CD4^+^CD28^+^ subpopulation in IC and PB samples. The MFI of CD28 in CD4^+^CD28^+^ subset was significantly lower in IC sample (p = 0.01, Wilcoxon signed rank test). **b** Representative flow cytometry histogram showing CD28 expression in IC (*plain line*) and PB (*dotted line*) samples

CD4^+^ CD28^null^T-cells measured as a percentage of CD4 T cells were significantly higher in IC samples (3.7 %, 0.9–8.5) than in the PB (2.9 %, 0.5–6.6) (p < 0.0001) (Fig. 1). Moreover, we evaluated the mean fluorescence intensity (MFI) of CD28 in CD4^+^CD28^+^ subpopulation. The expression of CD28 on the cell surface was significantly reduced in IC samples (median 310, IQR 240–368) compared with PB (median 338, IQR 248–387, p = 0.01) (Fig. 2).

We studied the association between levels of CD4^+^CD28^null^ T-cells and baseline characteristics of the population. As discussed below, pain to balloon time and CK levels were found associated with this subpopulation.

The proportion of CD4^+^ and CD8^+^ expressing the early activation marker CD69 was similar in IC and PB (Table 2). No differences were found in CD3^+^CD56^+^ T cells between PB and IC blood.

We assessed the frequency of CD4^+^CD25^bright^Foxp3^+^ regulatory T-cells (Treg) among IC and PB samples and no significant differences were found (Table 2).

#### NK cells

**Fig. 3:**
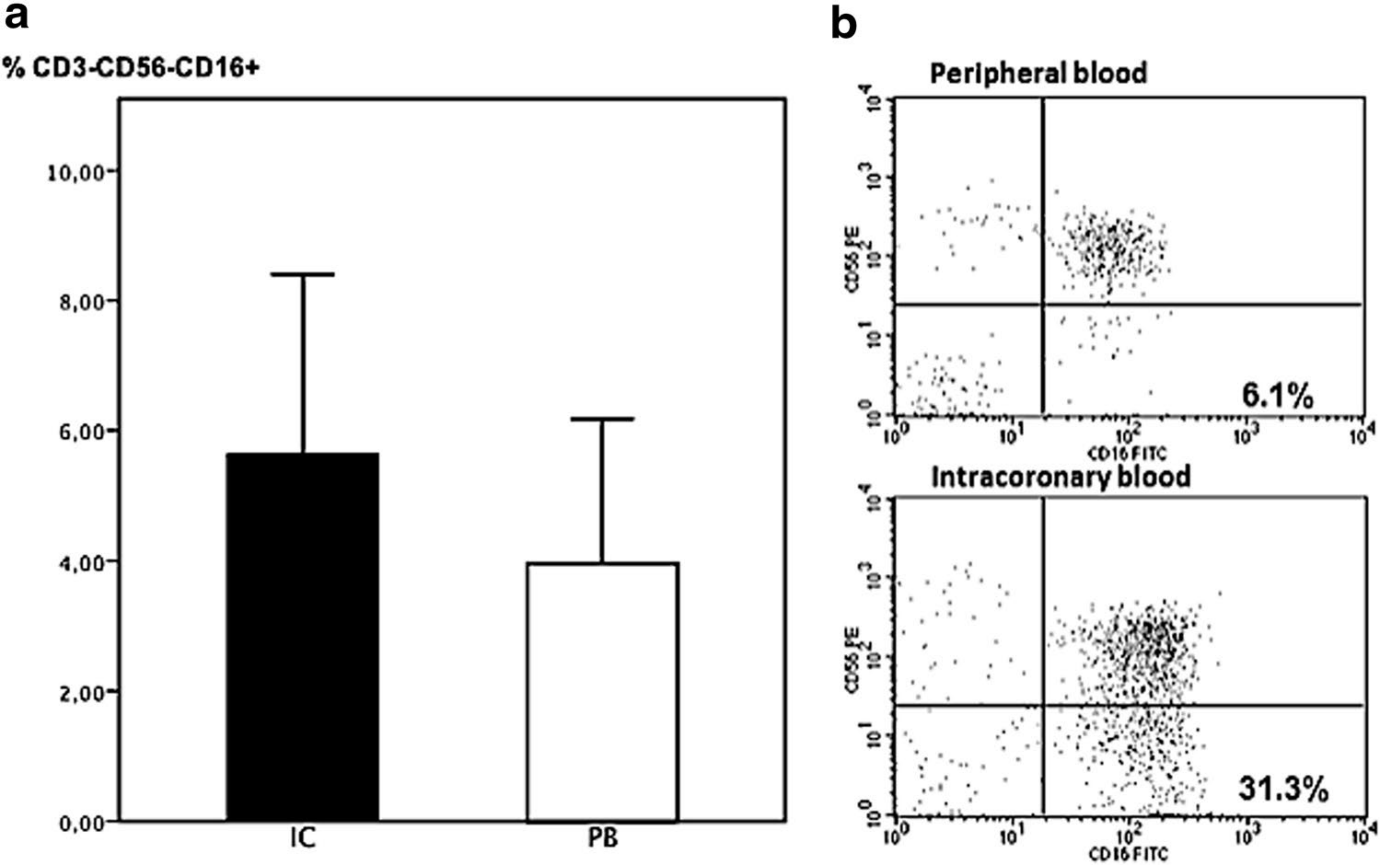
**a** CD3-CD56-CD16^+^ NK cell subset in IC and PB samples. The frequency of this NK cell subset was significantly higher in IC than PB (p = 0.006, Wilcoxon signed rank test). **b** Representative dot plot showing an increased percentage of CD3^−^CD56^−^CD16^+^ cells in IC vs. PB blood

We studied the frequencies of three major NK subpopulations: CD3^−^CD56^+^CD16^+^, CD3^−^CD56^++^CD16^−^, CD3^−^CD56^−^CD16^+^. These subpopulations were expressed as a percentage of total NK. No significant differences were found in total NK cells, CD3^−^CD56^+^CD16^+^ cells and CD3^−^CD56^++^CD16^−^ cells between PB and IC blood. However, the unusual subpopulation CD3^−^CD56^−^CD16^+^, was markedly higher in IC compared with PB samples (median 5.6 %, IQR 3.3–9.2 versus median 3.9 %, IQR 2.8–7.8, respectively, p = 0.006) (Fig. 3). Similar differences were also found when we compared the NK populations as a percentage of total lymphocytes.

#### B cells

The relative number of B cells (CD19^+^) was significantly lower in IC blood in comparison with PB. The median (IQR) of B cells was 10.3 % (4–13.2) in IC sample and 11 % (5.9–15.7) in PB sample (p = 0.008).

### Lymphocyte subsets according to prognostic clinical variables

We analyzed the distribution of the mayor lymphocyte populations in patients with more or with less than 6 h of evolution of STEMI (pain to balloon time) and with the peak creatine kinase (CK) level as infarct size estimation.

**Fig. 4:**
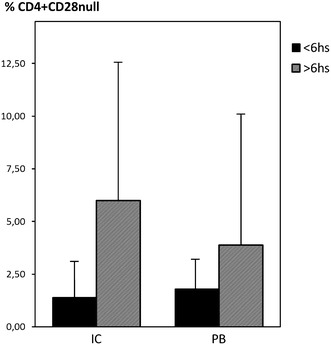
Frequency of CD4^+^CD28^null^ T cell subset according to the time of evolution of STEMI. In both, intracoronary microenvironment and PB, CD4^+^CD28^null^ T-cells were preferentially expanded in patients with more than 6 h of evolution of STEMI

As shown in Fig. 4, CD4^+^CD28^null^ T-cells in IC samples were preferentially expanded in patients with more than 6 h of evolution (n = 16) of STEMI (median frequency 5.9 %, IQR 3.9–12.1) compared with patients with less than 6 h of evolution (n = 17) (median frequency 1.4 %, IQR 0.6–3.62; p = 0.005 versus >6 h). In PB samples, we also found significantly higher frequencies of this CD4^+^ subset in the group with more than 6 h of evolution (median 3.8 %, IQR 2.5–9.5 versus median 1.7 %, IQR 0.3–3.2, respectively, p = 0.03) (Fig. 4).

Moreover, 11 (73 %) patients with more than 6 h of evolution of STEMI had more than 4 % of CD4^+^ CD28^null^ T-cells comparing with 4 (27 %) patients with less than 6 h (p = 0.012). When we evaluate very high frequencies (≥10 %) of CD4^+^ CD28^nullT−cells^ in IC and PB samples, all patients belong to the >6 h group (n = 6, p = 0.007 and n = 4, p = 0.044 respectively).

All other lymphocyte subsets, including T, B, and NK cells, were unaffected by the time of evolution of STEMI.

To study the association between lymphocyte subsets and infact size, patients were divided in two groups according to the median of CK levels (CK >than the median and CK ≤than the median).

The frequency of CD4^+^CD28^null^ T cells in IC blood was significantly higher in the group of patients with CK higher than the median as compared with the group of patients with CK lower than the median (median 5.9 % IQR 3.2–10.4 vs median 1.6 % IQR 0.6–5 p = 0.02) The same association was seen for PB (median 3.9 % IQR 2.1–8.8 vs median 1.3 % IQR 0.2–3.5 p = 0.04). Additionally, we found that the frequency of CD3^+^CD56^−^CD16^+^ NK cells was higher in the group of patients with higher CK levels in both IC (median 7.9 % IQR 4.1–10.2, vs median 3,6 % IQR 2.5–6.7 p = 0.03) and PB (median 7 % IQR 3.4–8.9 vs median 3.3 % IQR 2–4.5, p = 0.02).

No relationship with other clinical, hemodynamic or humoral parameters for CD4^+^CD28^null^ or other lymphocytes was noted.

### Intracellular cytokines in intracoronary and peripheral blood

**Fig. 5:**
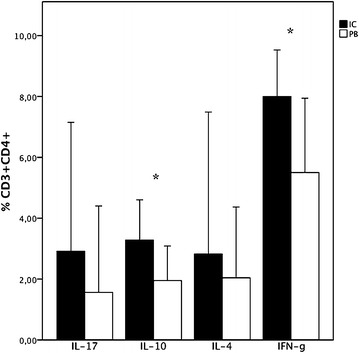
Percentage of CD3^+^CD4^+^ producing intracellular cytokines. The frequencies of CD3^+^CD4^+^IFN-γ^+^ and CD3^+^CD4^+^IL-10 + cells were significantly higher in IC blood. (p = 0.005 and p = 0.014, respectively, Wilcoxon signed rank test)

The CD4^+^T-cells production of IFN-γ, IL-17, IL-4 and IL-10 was studied in 10 patients (Fig. 5). Intracellular cytokine analysis showed a significantly greater number of IFN-γ producing CD4^+^ T-cells in IC blood (median 8.0 %, IQR 6.1–9.3 versus median 5.5 %, IQR 4–7.7, respectively, p = 0.005). In addition, the number of CD4^+^ lymphocytes producing IL-10 was also higher in IC than in the PB samples (median 3.3 %, IQR 1.1–4.3 versus median 1.9 %, IQR 0.7–3.1, respectively, p = 0.014).

No significant differences were found in the levels of intracellular IL-4 and IL-17 between IC and PB samples.

## Discussion

Inflammatory and immunopathological processes play a crucial role in the progress and destabilization of atherosclerosis, where T-cells are one of the dominant cell types in human ruptured coronary atherosclerotic plaques (De Palma et al. 2006; van der Wal et al. 1994). An increased pro-inflammatory Th1 response and enhanced expression of activation markers are frequently reported in ACS (Mazzone et al. 1999; Ranjbaran et al. 2007; Methe et al. 2005).

However, it is still undetermined whether and to what extent markers of the adaptive response originate locally from the site of plaque rupture and/or the thrombi or reflect a systemic response. To address this question, we investigated different lymphocyte subsets and intracellular cytokine production in intracoronary blood of patients with STEMI and compare them with their PB counterparts.

We found that in STEMI patients, CD4^+^CD28^null^ T-cells are preferentially expanded in the intracoronary microenvironment, at the site of the ruptured plaque relative to PB. Moreover, the PB frequency of this subset in these patients is elevated relative to healthy individuals. Based on previous reports, CD4^+^CD28^null^ account for about 0.1 to 2.5 % of the CD4^+^T-cells in healthy individuals (Dumitriu et al. 2009). Our findings are in line with previous reports showing that circulating CD4^+^CD28^null^ T-cells are increased in patients with ACS and accumulate preferentially in unstable atherosclerotic plaques (Liuzzo et al. 2000, 2007; Zal et al. 2004).

It has been suggested that CD4^+^CD28^null^ T-cells may affect plaque instability in patients with ACS. This T-cell subset shows high pro-inflammatory and tissue-damaging properties, including an increased production of IFN-γ and perforin (Liuzzo et al. 1999, 2000; Zal et al. 2004). CD4^+^CD28^null^ cells are therefore considered to play an important role in the events leading to coronary artery plaque destabilization and ACS (Nakajima et al. 2002). Our findings on the preferential expansion of this T cell subset in the local intracoronary microenvironment, at close proximity to the culprit lesion in patients with STEMI, support this hypothesis.

Interestingly, we also found downregulation of CD28 on the CD4^+^T-cells at the site of the ruptured plaque, as indicated by a significant reduction in the CD28 MFI in CD4^+^CD28^+^T-cells in intracoronary blood compared to their counterparts in PB. The expression of CD28 has been reported to be influenced by several cytokines. While TNF-α induces the downregulation of CD28 (Bryl et al. 2005), IL-12 induces the re-expression of functional CD28 molecules on the surface of CD4^+^CD28^null^ T-cells (Warrington et al. 2003). These findings, together with the increased frequency of intracoronary CD4^+^CD28^null^ cells, are in line with the idea that this T cell subset may belong to the same lineage as classic CD4^+^CD28^+^ T-cells (Vallejo et al. 2004; Rizzello et al. 2006).

Time elapsed from symptoms onset to PCI is an important factor influencing mortality in STEMI (Grines et al. 1993). In this work we studied the relationship between this factor and different subsets of lymphocytes. Significantly higher frequencies of CD4^+^CD28^null^ T-cells were found when we use a cutoff of 6 h of evolution. This cutoff was chosen because it has been reported that at this time myocardial salvage is minimal (Gersh and Anderson 1993). This observation was seen in both, IC and PB samples, although the expansion of CD4^+^CD28^null^ T-cells in patients with more than 6 h of evolution occurs preferentially in the intracoronary compartment. This association could be related to thrombus age or myocardial damage. To our knowledge, this is the first investigation of the relationship between pain to balloon time and the frequency of different lymphocyte subsets during the acute event.

Additionally, CD4^+^CD28^null^ were also increased in high CK levels group. As it is well known, CK level is a good predictor of infarct size (Kahn et al. 1979). Thus, according to the above results, we suggest that the higher the levels of CD4^+^CD28^null^ T-cells, the more intense the myocardial damage that may occur.

Little is known about the role of NK cell subsets in human atherosclerosis and plaque destabilization. Experimental evidence in animal models suggests that these cells play an important role in accelerating lesion development. This is achieved by modulating, via the production of IFN-γ, the function of other more prominent immune cells, like T lymphocytes and macrophages, in the atherosclerotic lesion (Rogers et al. 2008; Nakai et al. 2004; Whitman et al. 2004). Recently, it has been demonstrated that total NK and invariant NKT cells were significantly reduced in PB of patients with STEMI compared with healthy controls, which could suggest recruitment to the myocardial infarction (Liu et al. 2011). NK cells comprise 10–15 % of all peripheral lymphocytes and their identification is based on the expression of CD3, CD56 and CD16 (Orange and Ballas 2006). Three distinct NK cell subsets can be identified using these cell surface markers: CD3^−^CD56^++^CD16^−^, CD3^−^CD56^+^CD16^+^ and CD3^−^CD56^−^CD16^+^ (Cooper et al. 2001a, b; Montaldo et al. 2013).

In our study, we could not show an increase on total NK cells in IC compared with PB. However, CD3^−^CD56^−^CD16^+^ lymphocytes were expanded in intracoronary blood. This unusual NK cell subset has been found augmented in HIV (Mavilio et al. 2005), chronic hepatitis C virus (Gonzalez et al. 2009), tuberculosis infection (Barcelos et al. 2008) and Chagas disease (Vitelli-Avelar et al. 2006), but nothing is known about the participation of this subset in ACS. These NK lymphocytes, like CD4^+^CD28^null^ T-cells, might represent a key factor in the pathogenesis of plaque instability. Interestingly we found that CD3^−^CD56^−^CD16^+^ lymphocytes were also increased in the high CK level group.

The role of the CD3^−^CD56^−^CD16^+^ subset is unclear and controversial (Gaddy and Broxmeyer 1997), (Sondergaard et al. 2000). Bjorkstrom et al. (Bjorkstrom et al. 2010) reported that CD3^−^CD56^−^CD16^+^ NK cells are increased in chronic infected patients and have a lower cytotoxic capacity. They produce lower amounts of cytokines and degranulate to a lesser extent compared to CD56^+^ NK cells. However, they have an increased capacity to produce pro-inflammatory chemokines. Similar results were obtained in settings of chronic immune activation (Fuller and Zajac 2003). It is possible that similar loss of function occurs in STEMI.

Th1 cytokines have been associated with coronary disorders. IFN-γ, the main Th1 cytokine is produced by the majority of T-cells in human atherosclerotic plaques (Leon and Zuckerman 2005). It has been shown that IFN-γ may destabilize plaques through inhibition of smooth muscle cell proliferation, reduction of collagen synthesis and increased production of matrix metalloproteinase yielding rupture-prone plaques (Amento et al. 1991; McLaren and Ramji 2009). Previous studies have demonstrated a marked elevation in the percentage of peripheral circulating Th1 cells in ACS patients (Leon and Zuckerman 2005; Methe et al. 2005; Tedgui and Mallat 2006). Our results show that Th1 cells are increased in the intracoronary milieu. This could be due to migration of cytokine-producing T-cells to the site of inflammation or may reflect cytokine production by intraplaque T-cells that move to the intracoronary blood. Additionally, our study shows that CD3^+^CD4^+^IL-10^+^ cells were also expanded in the intracoronary blood. IL-10 is mainly produced by Th2 cells and Tregs and inhibits different aspects of the immune response, including Th1 cytokine production, antigen presentation and antigen-specific T cell proliferation (Tedgui and Mallat 2006). It has been reported that ACS patients have higher concentration of IL-10 in plasma than healthy controls (Malarstig et al. 2008). Moreover, the magnitude of IL-10 elevation correlates with the extent of systemic pro-inflammatory activity, based on plasma concentrations of CRP and IL-6 (Malarstig et al. 2008). Additionally, simultaneous elevation of IL-6 and IL-10 levels was associated with worse clinical outcomes in STEMI patients (Ammirati et al. 2012). Our findings of higher levels of CD3^+^CD4^+^ cells that produce IL-10 and IFN-γ in the intracoronary microenvironment is in line with these reports and suggest that the Th1 response elicited at the site of inflammation must be modulated by a regulatory T cell response.

### Limitations of the study

The main limitation of the study is the small number of patients included. Additionally, an important consideration for the in vivo analysis of intracoronary blood is that crossing the occluded coronary artery with a guidewire may result in partial restoration of epicardial blood flow. This may result in changes of the local frequencies of lymphocytes subpopulations. However, this phenomenon would play against our results because dilution with systemic blood would reduce rather than augment the significantly differences observed.

## Conclusions

To the best of our knowledge, this is the first report of detailed immunophenotypic characterization of lymphocyte subsets by flow cytometry in the intracoronary milieu, at the site of ruptured plaque in patients with STEMI in comparison to systemic levels. Coronary levels of immune cells with a proinflammatory profile at the site of plaque rupture were increased relative to their systemic counterparts. Moreover, we observed a positive correlation between the time elapsed from the onset of symptoms to PCI and the proportion of the CD4^+^CD28null T-cell population in IC and PB. Understanding the immunological processes underlying the pathogenesis of acute coronary syndromes may open new avenues for therapeutic intervention to prevent complications of atherothrombosis.

## Methods

### Study population

We prospectively evaluated 33 consecutive patients with STEMI who underwent primary angioplasty for reperfusion in the Cardiology Department of the University Hospital of Montevideo.

The inclusion criteria were: (a) chest pain lasting more than 30 min, (b) ST-segment elevation ≥2 mm in at least two contiguous leads, (c) less than 24 h of evolution, (d) occlusion of an infarct-related artery (TIMI flow <2) and (e) clinical indications for PCI and thromboaspiration.

Exclusion criteria were: (a) evidence of inflammatory or infectious disease, malignancies, immunologic or hematological disorders; and (b) treatment with anti-inflammatory drugs other than aspirin.

Demographic data, classic risk factors, angiographic findings, and medical treatments at the time of coronary angiography were carefully recorded for all patients.

#### Ethical statement

The study protocol was approved by the Institutional Ethics Committee. All participants gave and signed informed consent, and the principles of the Helsinki Declaration were respected.

### Blood samples

Coronary angiography was performed according to standard techniques. Anticoagulation was achieved with unfractioned heparin (70 UI/kg) administered after sheath insertion and clopidogrel (300 mg) and aspirin (325 mg) were given to all patients before blood sampling.

Arterial peripheral blood samples (PB) were obtained from the femoral or radial artery after sheath insertion. The infarct-related coronary artery was initially catheterized using a 6–7 French guiding catheter. The lesion was subsequently wired with a 0.014 inch guidewire. A monorail aspiration catheter (Export, Medtronic) was advanced up to the thrombus, and intracoronary blood (IC) sample was collected from the distal end of the coronary lesion. PCI was then performed according to standard techniques.

PB and IC blood samples were processed for lymphocytes subsets distribution and intracellular cytokine production at the Laboratory of Flow Cytometry and Molecular Biology within 12 h of being obtained.

### Flow cytometric analysis for lymphocytes subsets

Blood samples were immunostained with the following panel of antibodies: APC-conjugated anti CD3, FITC-conjugated anti CD4, PE-conjugated anti CD69, PECy5-conjugated anti CD8, PE-conjugated anti CD28, PerCP-conjugated anti CD3, FITC-conjugated anti CD16, PE-conjugated anti CD56, PerCP-conjugated anti CD19, FITC-conjugated anti CD25, PE-conjugated anti Foxp3 and APC-conjugated anti CD4 (all reagents from BD Pharmingen, San Diego, USA). Optimal antibody concentrations were previously defined by titration. For intracellular Foxp3 staining, samples were first stained for CD4 and CD25, then fixed and permeabilized with human Foxp3 buffer set (BD Pharmingen, San Diego, USA) according to manufacturer´s protocols. Briefly, cells were washed twice with permeabilization buffer and then incubated with anti-human Foxp3 at room temperature for 30 min in the dark, before being resuspended in PBS and analyzed.

Flow cytometry data was collected on a FACSCalibur flow cytometer equipped with two lasers (Becton–Dickinson, Oxford, UK). For data acquisition and analysis CellQuestPro software (Becton–Dickinson) was used.

### Analysis of intracellular cytokines

Intracellular flow cytometry was utilized to determine the percentage of IFN-γ, IL-4, IL-17 and IL-10-producing lymphocytes within the CD4^+^subset in 10 patients, in both IC and PB samples. Briefly, heparinized whole blood was diluted 1:1 with RPMI medium (Sigma-Aldrich Co., St Louis, MO, USA), and cells were stimulated for 5 h at 37 °C, in 5 % CO2 atmosphere with 0.5 ng/ml ionomycine (Sigma-Aldrich) and 1 γg/ml phorbol myristate acetate (PMA; Sigma-Aldrich) in the presence of 5 mg/ml Brefeldin A (Sigma-Aldrich) to induce cytokine production (Grille et al. 2010). Lymphocytes were then stained with anti-human CD4 and anti-human CD3 (BD Pharmingen, San Diego, USA). Red blood cells were lysed with FACS Lysing Solution (BD Biosciences), and lymphocytes were permeabilized with FACS Permeabilizing Solution (BD Biosciences). To detect intracellular cytokines, cells were stained with the following cytokine-specific antibodies: anti-human IL-17-PE, IFN-γ-APC, IL-4-PE and IL-10-APC (BD Pharmingen, San Diego, USA), washed, and fixed with 1 % paraformaldehyde. Data acquisition and analysis was performed as previously described. A gate was set on CD4^+^CD3^+^ lymphocytes and at least 5000 cells were counted and evaluated for the expression of IFN-γ, IL-17, IL-4 and IL-10.

### Statistical analysis

Standard descriptive and comparative analyses were done in SPSS 18.0. The lymphocyte subsets frequencies were expressed as median, standard error and range; the remaining variables were expressed as mean ± SD.

The Kolmogorov–Smirnov test showed that lymphocyte subset frequencies did not follow a normal distribution thus, nonparametric tests were used. The Wilcoxon signed rank test for paired data was used for comparison between IC and PB samples. The Mann–Whitney U test was used to estimate possible differences between patients with less or more than 6 h of evolution of STEMI and between patients with more or less than the median CK levels. Proportions were compared using the Chi square test. Statistical significances were set at a two-tailed p-value of <0.05.

A cutoff of 4 % CD4^+^ CD28^null^ T-cells was chosen to define patients with low or high frequencies of these cells, because 4 % represented the 90th percentile of distribution in healthy subjects (median 0.73 %, range 0.05–4.94 %) (Martens et al. 1997). Moreover, patients with ≥10 % were considered to have very high frequencies of these cells, because this value was more than tenfold higher than the median value in the normal population.

## Abbreviations

PB: peripheral blood
IC: intracoronary
STEMI: ST-segment elevation myocardial infarction
NK: natural killer
Tregs: regulatory T cells
PCI: percutaneous coronary intervention
WBC: white blood cells
ACS: acute coronary syndrome
MFI: mean fluorescence intensity
IQR: interquartile range
